# Comprehensive power quality performance assessment for electrical system of a nuclear research reactor

**DOI:** 10.1038/s41598-023-36692-2

**Published:** 2023-06-19

**Authors:** Asmaa M. Elsotohy, Ahmed Mohammed Attiya Soliman, Ahmed S. Adail, Ayman A. Eisa, El-said A Othman

**Affiliations:** 1 Nuclear Safety and Radiation Emergencies Research Department, NCRRT, Egyptian Atomic Energy Authority, Cairo, Egypt; 2 Department of Electrical Engineering, Faculty of Engineering, Al-Azhar University, Nasr City, Cairo, 1427 Egypt; 3 Department of Fuel Technology, Hot Laboratory Centre, Egyptian Atomic Energy Authority, Cairo, Egypt

**Keywords:** Electrical and electronic engineering, Engineering

## Abstract

Studying the power quality (PQ) is an essential issue to ensure the safe and accurate operation of sensitive equipment particularly for nuclear installations. Assessment of PQ involves collecting and analysing data resources and then evaluating it with reference to PQ standards. There are many alternatives for PQ and it is difficult to make an appropriate selection among them in the existence of their multiple criteria which are usually conflicted. So this selection subject can be classified as a Multi Criteria Decision Making (MCDM) problem. To do so, a reliable and scientific method for studying and evaluating the overall system PQ is required. This study aims to assess performance of PQ for the electrical power system at a Nuclear Research Reactor (NRR) during a certain period using multiple measures for the most decisive PQ phenomena. It focuses on a number of the most important PQ phenomena namely frequency fluctuation (deviation), unbalances of current and voltage, current and voltage harmonic distortion, flicker and power factor. After combining all results into six samples (alternatives), the criteria weights are determined based on an objective method for weighting which is called CRITIC method. Then, the alternatives are ranked using compromise MCDM method-VIKOR method. The obtained results are analyzed and discussed to evaluate performance of NRR electrical system from the PQ view. It showed that the compromise solution that obtained by CRITIC-VIKOR can be a guide to facilitate the PQ evaluation of nuclear installation electrical system. Also, it can empower the operators with the benefits of benchmarking and monitoring a single index instead of several indices. Moreover, it is very useful for helping stakeholders to understand how the PQ performance changes under a certain operating condition of the facility. Finally, it is can be considered as a good model to weight each PQ phenomena and identify the time intervals for best and worst total PQ in NRR.

## Introduction

Electrical power systems are exposed to power quality (PQ) problems and disturbances. PQ refers to multiple electromagnetic phenomena that characterize the power system current and voltage at a given location and time. The increasing existence of electronic equipment that can be highly sensitive to these phenomena, or that can be a source of electromagnetic disturbances, has heightened and increased the interest with PQ in recent years^1^.

In response to the complex PQ problems in electrical system, PQ evaluation is a very important issue to identify the PQ performance, and its obtained results can be utilized as a reference in the PQ field of electrical power system. Usually, the total PQ performance is derived depending on multiple indices^2^. To get a total PQ index, it is required to assess and analyze the data of PQ, and its main task is to get a single index by ways of merging and weighting of multiple PQ indicators (indices)^3,4^—that is, to sort and rank the samples of PQ which contain multiple PQ indicators.

Till now, there is no standard documented method that can be used by operators of the electrical systems to benchmark and evaluate the overall network PQ performance^5^. Some of the published researches tried to facilitate the PQ performance evaluation through a number of different methodologies. However, a number of the current methods for evaluation of PQ have not taken into consideration the correlation among indices of PQ, or there are some weakness and shortcomings in the methodologies of weighting and indexing^6^. The artificial neural network method that was presented in^7,8^ as a technique to get a total PQ index needed to a huge amount of sample data to train and test the network. The fuzzy evaluation method suggested in^9,10^ depend on experience too much and is too subjective determining membership function and obtaining weights. Nourollah et al.^11^ obtained a global index for sorting and ranking PQ based on data mining. Both the discrete and continuous PQ disturbance are considered and based on the method of incorporation and normalization a unified PQ index (UPQI) is obtained. Moreover, the PQ data at 313 distribution sites in Iran was monitored and are used to classify the indices for different loads types in the distribution network (DN), but this method depended on the expert’s experience.

Power transformers are of vital importance for the stable operation of power system. A fuzzy-AHP model is used to evaluate the transformer performance based on its different parameters^12^. The Multifunctional grid-tied inverters (MFGTIs) have been paid much attention to handle the commonly concerned power quality issues of the microgrids. Therefore, paper^13^ proposed an objective-oriented model based on AHP theory to enhance the power quality of the microgrid by optimal utilization of the limited and valuable MFGTI capacity. The proposed model on this study is applied on a feeder of a microgrid with two identical 10-kVA DGs. Ref^14^ proposed AHP model to combine the harmonics, unbalance and voltage sag performances in one combined index. Based on this model, The separate and cumulative PQ performances of a 295-bus generic distribution network were evaluated, compared and demonstrated using heat maps. In^15^ the AHP model was used for evaluating and benchmarking the PQ performance of grid-integrated renewable energy systems. The proposed approach was verified on a grid-integrated PV-based DG system in MATLAB/Simulink. Lee et al.^16^ proposed a UPQI based on the ideal AHP which can be utilized to evaluate DN performance comprehensively mainly for planning purposes of electrical system. Mohit et al.^17^ also presented AHP to compute a PQ index. The proposed technique is applied on a DN of two buses with a high emissions degree of harmonic induced by the both load and utility nonlinearity. The limitation of traditional AHP methodology is that usually construction of a judgment matrix depends on the expert’s opinion.

Ref^18^ proposed an index system of power quality and an evaluation method based on power customer perception information. Based on Entropy Weight (EW) method, power quality was evaluated and analyzed for a power grid in China. The AHP-EW combination weighting method and the improved Technique for Order Preference by Similarity to Ideal Solution (TOPSIS) method proposed in^19^. Voltage flicker, total harmonic distortion for voltage, three-phase voltage unbalance, voltage deviation, voltage fluctuation and frequency deviation are taken into consideration. Moreover, the PQ data measured for a large wind farm in China at the five main substation busbar nodes of this farm. The five substation nodes were: 10 kV busbar of substation 1, 110 kV busbar of substation 2, 10 kV busbar of substation 3, 35 kV busbar of substation 4 and 10 kV busbar of substation 5. Five samples of PQ are obtained by analyzing the measurements on each substation and converting them to a single sample. This methodology was suitable for various evaluation scenarios, particularly in the case of multiple indices. Hongtao Shi et al.^20^ presented a comprehensive PQ assessment method to identify the objective dynamic weight of PQ indicators of a microgrid using Criteria Importance Through Intercriteria Correlation (CRITIC) method for both single and multi-node. This research is suitable for PQ assessment in microgrid systems but it can’t make a ranking for the total power quality.

In the current study, a comprehensive PQ evaluation of NRR electrical system measurements based on Multi Criteria Decision Making (MCDM) is presented. firstly, the objective weights for each PQ phenomenon are obtained using the CRITIC method. Then based on VIKOR ranking method, a grade for each alternative is calculated. A comparison and analysis of the obtained results is explained in details to evaluate the total PQ for this nuclear facility. The main objective of this study in assessing the total PQ is achieved. The novelty of this study is that this is the first time to use the combination of these two methodologies in the PQ field and applying them on a nuclear facility.

The parts of this paper are organized as follows: After the introduction, a brief description of the Nuclear Research Reactor (NRR) electrical power system is covered in “General description for electrical power system of the NRR” section. “Analysis of NRR power quality problems” section explains and presents an analysis of PQ phenomena and indices of NRR which will be utilized on the proposed approach formulation. “Research methodology” section explains the study methodology in detail, and “Applying the proposed research methodology on the measurements results of the NRR electrical system” section presents the case study, Finally, “Conclusion” section contains conclusion of the paper and presents the future work followed by data availability then a list of references is written in the end.

## General description for electrical power system of the NRR

For nuclear facilities, the system of electrical power is considered one of the most important systems. Providing sufficient power with the required quality to nuclear facility equipment and systems is a very important issue to ensure reliable and safe operation for all nuclear facilities.

The electrical loads of NRR have been classified according to the following categories:

- Class ‘A’ loads: are those loads essential from a safety point of view; they required uninterruptible AC power supply (UPS). The capacity of UPS is 15 KVA. This capacity meets all class ‘A’ required load demands and conditions with autonomy of 30 min.
- Class ‘B’ loads: are loads whose reconnection to the system is convenient in order to increase their availability after interruption of electrical supply from the external lines. Class ‘B’ loads are fed by two sources, the normal power supply and the power plant. The power plant has two diesel generators design to furnish AC power adequate for supplying class ‘B’ and the uninterruptible power system in case the external lines are unavailable. The capacity of plant is two generators with 300 KVA for each.
- Class ‘C’ loads: they admit interruption the supply for definite time. They fed from the normal power supply.

**Figure 1:**
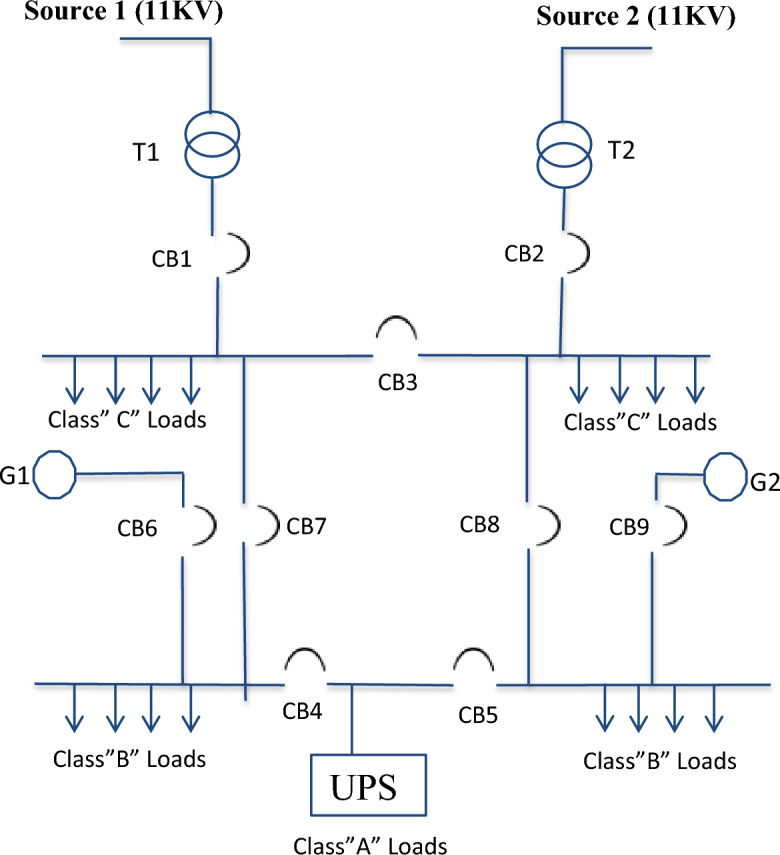
A single-line diagram for NRR electrical system.

As shown in Fig. 1, the electrical power system of NRR is fed from two different substations by two independent sources (source 1, source 2) at a medium voltage level to guarantee good reliability. Each source feeds a separate transformer through a circuit breaker (CB). The normal power supply is capable of starting and operating all required loads and the transformers are identical, each of them (primary voltage 11 kV, 50 Hz, secondary voltage 0.4/0.231 kV, connected group Dy 11) has 100% of the total sum of individual maximum demands. Each transformer has a capacity of 2000 kVA, which is the power required for the NRR.

## Analysis of NRR power quality problems

NRR is affected by abnormal conditions of the electrical power system and this leads to defects on its electrical system such as:

- Some component failure such as capacitors, electronic cards, etc.
- Circuit Breaker tripping and transformer failures.
- Noise, excessive vibration, overheating, and burning out for motors.
- Damage to a number of ballasts and lamps.
- Operation irregularly for sensitive equipment.
- Loss of electrical power and hence nuclear reactor shutdown.
- Errors and malfunction signals lead to nuclear reactor shutdown.
- Performance for system of electrical power is bad.

Electrical PQ is mainly affected by different disturbances such as disturbances originating from the utility feeding systems, disturbances originating from consumers’ networks and devices, nonlinearity of devices and loads, or selecting unsuitable sites for transmission and distribution lines.

The PQ performance usually is determined based on different PQ phenomena. Among the most important PQ phenomena are frequency deviation (F_dev), voltage unbalance (V_Un), current unbalance (I_Un), current harmonic distortion (ITHD), voltage harmonic distortion (VTHD), long-time flicker (P_lt_), short-time flicker (P_st_) and power factor (PF). This section presents in detail a brief definition of these PQ phenomena and analysis for PQ data measurements of incoming feeder from distribution centre to the nuclear research reactor for a period of 50 h with an interval of 5 min at a certain operating condition. The max and min obtained values are shown in Table 1. Also, a comparison of measured values with standard acceptable limit is presented in this section^1,21^.

**Table 1:** Maximum and minimum values for NRR measurement results.

	V_Un %	I_Un %	VTHD %	ITHD %	Plt %	Pst %
Min. value	0.01	0.32	0.61	1.78	0	0
Max. value	0.14	6.62	1.5967	13.3567	0.4533	1.0343
Standard limit	2	5	5	15	0.8	1

### Voltage unbalance

Unbalance is defined as the percentage ratio of the negative sequence component magnitude to the positive sequence component magnitude. This definition can be applied to both current and voltage. Usually, for a three-phase service, the unbalance of voltage is less than 5%. The current unbalance can be higher, especially when there are single-phase loads on the system.

Unbalanced single-phase loads are the main source of small voltage unbalance (less than 2%) on a three-phase circuit. Also, voltage unbalance can result from capacitor bank anomalies. Severe voltage unbalance (more than 5%) can be due to conditions of single-phasing as an open protective device upstream of the monitoring point.

**Figure 2:**
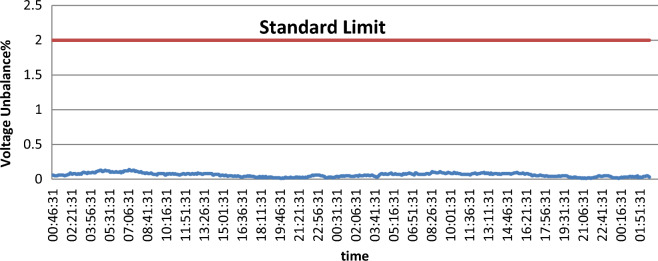
Voltage unbalance.

**Figure 3:**
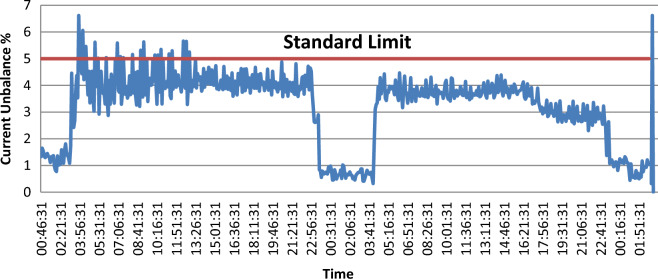
Current unbalance.

It is shown in Fig. 2 that the voltage unbalance percentage does not exceed the IEEE acceptable limit of 2%, which most devices and equipment can tolerate. According to the ANSI C84. 1–1995 standard, the maximum current unbalance value is 5%. Figure 3 shows that the current unbalance is higher than the voltage unbalance and exceeds the standard limit in some points.

### Voltage fluctuations (flicker)

The impact of voltage fluctuation on the intensity of lighting produces the flicker phenomenon. With another explanation, the fluctuation of voltage is an electromagnetic phenomenon, and this phenomenon leads to undesirable results in lighting which is called flicker. One of the causes of voltage fluctuations or flickers is the loads which can exhibit continuous and rapid variations. Characterizing the voltage flicker severity with respect to human visual perception sensitivity is the present industry practice. In utility transmission and distribution systems, Arc furnaces and welders are considered the most common sources of voltage fluctuations. According to IEC, the disturbance severity is described as the short-term severity (${p}_{st}$), which is measured over a time of ten minutes, and the long-term severity (${p}_{lt}$), which is calculated from ${p}_{st}$—values over a two hours interval, according to the following equation:

1 $$p_{lt} = \sqrt[3]{{\sum\limits_{i = 1}^{12} {\frac{{p_{{st_{i} }}^{3} }}{12}} }}$$

Generally, a ${p}_{st}$ less than 1 is not perceived by most people in normal environments.

**Figure 4:**
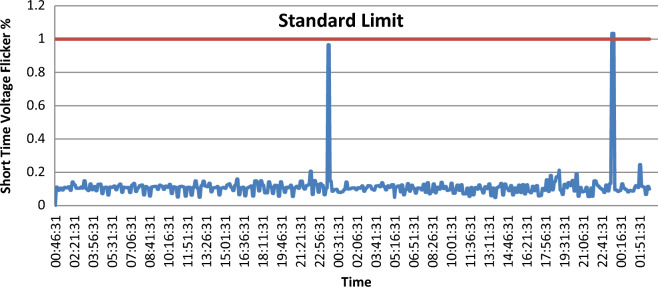
Short time voltage flicker (Pst).

**Figure 5:**
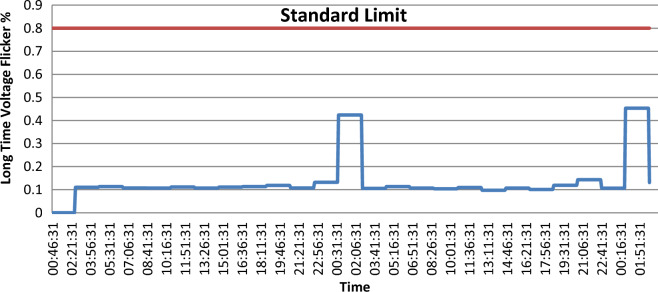
long time voltage flicker (Plt).

As shown in Fig. 4, throughout the whole survey ${p}_{st}$ exceeds the acceptable limits only on one point. On the other hand, as shown in Fig. 5, ${p}_{lt}$ doesn’t exceed the IEEE limit.

### Waveform distortion

It is a steady-state deviation from the ideal sinusoidal waveform of power frequency. The main types of waveform distortion are notching, harmonics, interharmonics, noise and DC offset.

*Harmonics* are sinusoidal currents or voltages having frequencies that are integer multiples of the fundamental frequency. Distortion of harmonic originates due to the loads and devices nonlinearity in the power system. Harmonic resonance is one of the major problems associated to harmonic disturbances as it can cause equipment malfunction or damage. There are other effects of harmonics such as increased losses, equipment overloading, and sometimes equipment malfunction. The most commonly used harmonic indices are the total current harmonic distortion (${THD}_{I}$) and the total voltage harmonic distortion (${THD}_{v}$).

${THD}_{v}$ Is obtained from Harmonic voltage ${v}_{h}$ and it can be calculated according to the following equation:

2 $$THD_{V} = \frac{{\sqrt {\mathop \sum \nolimits_{h = 2}^{\infty } V_{h}^{2} } }}{{V_{1} }}*100\%$$

${THD}_{I}$ Is obtained from Harmonic current ${I}_{h}$ which is result from the nonlinear devices operation on the power system and it can be calculated according to the following equation:

3 $$THD_{I} = \frac{{\sqrt {\mathop \sum \nolimits_{h = 2}^{\infty } I_{h}^{2} } }}{{I_{1} }}*100\%$$

**Figure 6:**
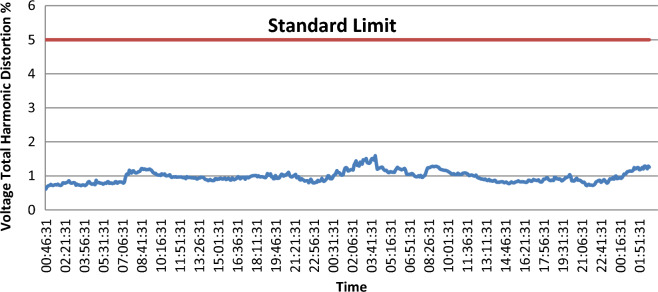
voltage total harmonic distortion (VTHD).

**Figure 7:**
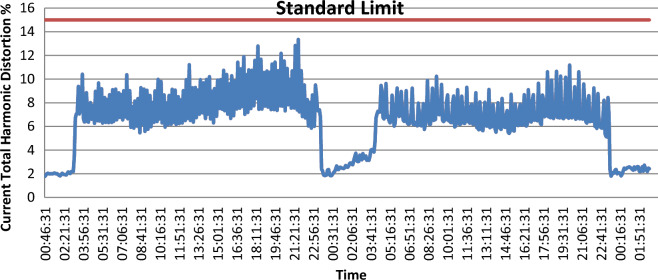
Current total harmonic distortion (ITHD).

As shown in Fig. 6 the ${THD}_{V}$ levels don’t exceed the acceptable tolerance limit of 5% set by standard of IEEE. On the other hand, ${THD}_{I}$ levels are close to the acceptable tolerance of 15% set by the standard of IEEE And as shown in Fig. 7.

### Power frequency variation

**Figure 8:**
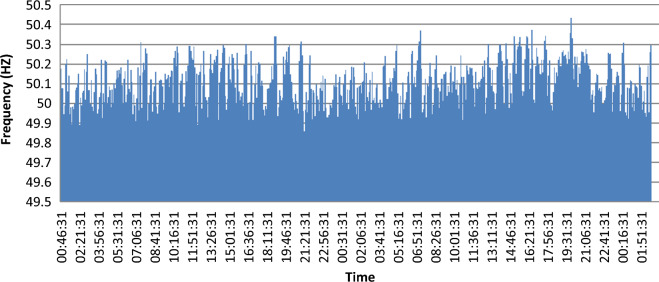
The Electrical Frequency.

It is the deviation of the system frequency its nominal value. The system frequency is related directly to the generator’s rotational speed supplying the system. Slight frequency variations happen when a dynamic balance between generation and load changes. The frequency shift duration and size influenced by the generator control system response to load changes and the characteristics of the load. Figure 8 shows the change of power frequency during the measurement period.

### Power factor

Power factor (PF) is a measure of how effectively you are using electricity. It is the ratio of active power to apparent power. A low PF indicates poor electrical power utilization, while a high PF benefits both the utility and the customer.

**Figure 9:**
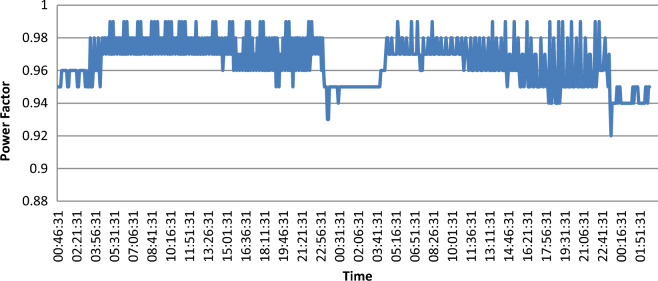
Power factor.

Under ideal conditions, voltage and current are “in phase” and the PF is “100%”. In the case of existing inductive loads (motors) in power circuit, a power factor less than 100% can occur. As shown in Fig. 9, during all measurement period, PF variations lie in the range of 100% to 92% and this present a very good PF.

All the PQ phenomena described above, reflect the PQ picture for NRR electrical system during a certain period. The main importance of this assessment and evaluation lies in the fact that the deviation of these PQ phenomena from the standard limit leads to a decrease in the system component’s useful life and an increase in the costs of operating. Where, the disturbance that led to the nuclear reactor shut down, presents losing of money. The amount of financial loss depends on the reactor fuel state and cost. Normally after reactor scram, it needs around one hour to restart, but need about 50 h to restart if it was at the end of fuel cycle due to the reactivity. From experience the disturbance cost of NRR is approximately (5000 $) at normal state^21^.

## Research methodology

**Figure 10:**
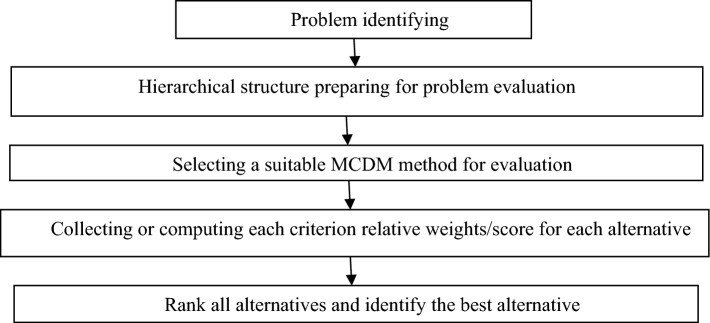
Flow chart for steps of the MCDM Evaluation method.

MCDM is an advanced research field that helps analysts and decision-makers and provides them with a wide range of scientific methods, which are suitable for complex economical decision problems^22^. MCDM refers to selecting, prioritizing, or ranking a set of alternatives under conflicting, independent attributes or criteria then it determines the appropriate alternative among several, alternatives. Figure 10 shows a flow chart for steps of the MCDM Evaluation method^23^.

Application of MCDM depends on the calculating of criteria weights that have importance for alternatives. The methodology of criteria weights estimation which depends on the decision maker’s opinions is a subjective weighting method (e.g. AHP, pairwise comparison method, Simple Multi-Attribute Rating Technique (SMART), SWING, SIMOS, Revised SIMOS, Delphi method, etc.) and this influenced by the experience and knowledge of decision-makers in the problem field and in the related fields.

On the other hand, the weights are determined on objective weighting methods (e.g. Mean Weight (MW), Statistical Variance procedure, Standard Deviation (SD), EW, (CRITIC), etc.) by solving mathematical models without taking into account any consideration for preferences of the decision-makers.

After identifying the criteria weights, there are a number of MCDM techniques (TOPSIS, WAPSAS, VIKOR, PROMETTHE, etc.) that focus on ranking for alternatives in the existence of conflicted criteria^24^.

In the current study, the selected method for weighting is the CRITIC method as it is based mainly on the analytical investigation for the matrix of evaluation to extract all contained information regarding the evaluation criteria. With another explanation, CRITIC method derived objective weights by quantifying the intrinsic information of each criterion^24^. The selected method for ranking is VIKOR as it is one of the most applied MCDM methods used for complex systems. It ranks and determines the best and worst choice among a number of alternatives in the existence of conflicted criteria^25^. Also, the VIKOR method achieves a compromise ranking by comparing the closeness degree with the ideal solution. The key feature of VIKOR method is that it minimizes the individual regret and maximizes the group benefits and so that its result can be accepted by decision-makers^26^. These two methods will be clarified in detail on the following sections as CRITIC-VIKOR can show results more realistic because of the differential weights assigned to criteria by the CRITIC method^24^.

### The CRITIC method

This method determines objective weights for criteria. In this methodology, the procedure of weight determination for criteria contains both criteria correlation and its standard deviation. To get the ${j}_{th}$ criterion weights, the following steps are followed^24^:

- *Step 1* Decision matrix formulation
  Build the main decision matrix $X={\left[{x}_{ij}\right]}_{m*n}$ containing *m* alternatives and *n* criteria, based on the different available data and required target, where ${x}_{ij}$ is a measure for the performance of ${i}_{th}$ alternative with respect to ${j}_{th}$ criterion.
- *Step 2* Decision matrix normalization
  It is a process to transform the values into standard scales depending on the worst and best value for each criterion, which range between 0 and 1 using Eq. 4 that presents the normalized matrix $({r}_{ij})$ of alternative $i$ with respect to criterion $j$ as follows:
  4 $$r_{ij} = \frac{{x_{ij} - x_{j}^{worst} }}{{x_{j}^{best} - x_{j}^{worst} }}$$
  where ${x}_{ij}$ is the actual value of alternative $i$ with respect to criterion $j$, ${x}_{j}^{best}$ is the best value of criterion $j$, and ${x}_{j}^{worst}$ is the worst value of criterion $j$.
- *Step 3* Standard deviation estimation for the normalized matrix.
  Standard deviation is an indicator of how much the value has deviated from the mean value. When the values are far from the mean value, a high standard deviation will be obtained, whereas a low standard deviation means that the values tend to be close to the mean.
  The standard deviation ${\sigma }_{j}$ of the ${j}_{th}$ criterion on the normalized matrix can be calculated based on the mean score of m alternatives ($\overline{{x }_{i}}$) using Eq. (5).
  5 $$\sigma_{j} = \sqrt {\frac{{\mathop \sum \nolimits_{i = 1}^{m} \left( {x_{i} - \overline{{x_{i} }} } \right)^{2} }}{m - 1}}$$
- *Step 4* Calculation of the correlation between pairs of criteria.
  A correlation matrix is a table that presents coefficients of correlation between variables. Each random variable (${x}_{i}$) in the normalized matrix is correlated with each of the other values (${x}_{j}$) as shown in Eq. (6) used to calculate the correlation between pairs of criteria (${\rho }_{jk}$). This lets the decision’s maker know which criteria pairs have highest correlation.
  6 $$\rho_{jk} = \frac{{\mathop \sum \nolimits_{i = 1}^{m} \left( {r_{ij} - \overline{{r_{j} }} } \right)\left( {r_{ik} - \overline{{r_{k} }} } \right)}}{{\sqrt {\mathop \sum \nolimits_{i = 1}^{m} \left( {r_{ij} - \overline{{r_{j} }} } \right)^{2} \mathop \sum \nolimits_{i = 1}^{m} \left( {r_{ik} - \overline{{r_{k} }} } \right)^{2} } }}$$
  where: $i$= 1, 2, …, *m*; $j, k$= 1, 2, …, *n*, and ${r}_{jk}$ Is the coefficient of correlation between the ${j}_{th}$ and ${k}_{th}$ criteria.
  By subtracting the correlation coefficient ${r}_{ik}$ from the number one ($1-{\rho }_{jk}$), a measure of the conflict created by criterion *J* with respect to the decision situation defined by the rest of the criteria can be calculated. Noting that, the conflict will decrease with the correlation coefficient increase.
- *Step 5* Determining the amount of information for each criterion.
  Equation (7) is used to identify the information amount in the ${j}_{th}$ index (${C}_{j}$), the larger index ${\mathrm{C}}_{\mathrm{j}}$ is the greater information amount contained in the index.
  7 $$C_{j} = \sigma_{j} {*}\sum\nolimits_{k = 1}^{n} {\left( {1 - \rho_{jk} } \right)}$$
- *Step 6* Determining the objective weight.
  Depending on ${C}_{j}$ the objective weight (${W}_{j}$) for each criterion can be obtained from Eq. (8).
  8 $$W_{j} = \frac{{C_{j} }}{{\mathop \sum \nolimits_{k = 1}^{n} C_{k} }}$$

### VIKOR method

The basics of this method were first offered by Yu^27^ and Zeleny^28^ later it is supported by Tzeng and Opricovic^29,30^. It is one of the applicable MCDM methodologies for solving optimization problems of complex systems that have multi-criteria. The VIKOR fundamental principle is to rank a number of alternatives and identify the best one between them^30^. It determines a compromise ranking list and the intervals for weight stability of the obtained compromise solution^31^.

The compromise ranking algorithm VIKOR has the following steps:

- *Step 1* Computing the worst value ${f}_{j}^{-}$ and the best value ${f}_{j}^{*}$ of all criteria and using these values to build the normalized matrix ${wx}_{new}$ using Eq. (10).
  9 $$\begin{aligned} & f_{j}^{ - } = min_{i} f_{ij} \\ & f_{j}^{*} = max_{i} f_{ij} \\ \end{aligned}$$
  10 $$wx_{new} = w_{j} \left( {f_{j}^{*} - \left. {f_{ij} } \right)/\left( {f_{j}^{*} - \left. {f_{j}^{ - } } \right)} \right.} \right.$$
  where ${f}_{ij}$ is the value of ${j}_{th}$ criterion for the alternative ${a}_{i}$ , $j = 1, 2, \dots , n$, and $i = 1, 2, \dots , m$ *for n* criteria and *m* alternatives.
- *Step 2* Determining the regret measure values (${R}_{i}$) and the utility measure values (${S}_{i}$) by the relations:
  11 $$R_{i} = max_{j} \left[ {w_{j} \left( {f_{j}^{*} - \left. {f_{ij} } \right)/\left( {f_{j}^{*} - \left. {f_{j}^{ - } } \right)} \right.} \right.} \right]$$
  12 $$S_{i} = \mathop \sum \limits_{j = 1}^{n} w_{j} \left( {f_{j}^{*} - \left. {f_{ij} } \right)/\left( {f_{j}^{*} - \left. {f_{j}^{ - } } \right)} \right.} \right.$$
  where,${w}_{j}$ is the $jth$ criterion weight (relative importance).
- *Step 3* Computing values of alternative ranking indicator (${Q}_{i}$), $i = 1, 2, \dots , m$, using Eq. (13)
  13 $$Q_{i} = v\left( {S_{i} - S^{ - } } \right)/\left( {S^{*} - S^{ - } } \right) + \left( {1 - v} \right)\left( {R_{i} - R^{ - } } \right)/\left( {R^{*} - R^{ - } } \right)$$
  where ${S}^{-}$ =${min}_{i}$ ${S}_{i}$, ${S}^{*}$=${max}_{i}$ ${S}_{i}$ and ${R}^{-}$ =${min}_{i}$ ${R}_{i}$, ${R}^{*}$=${max}_{i}$ ${R}_{i}$ and $v$ is the strategy weight of maximum utility for the group and its value usually equal 0.50.
- *Step 4* Get a ranking for the alternatives by making three ranking lists based on sorting the ${S}_{i}$, ${R}_{i}$, and ${Q}_{i}$ values in decreasing order.
- *Step 5* Suggest as a compromise solution by checking to what extent either the following two conditions are satisfied or one of them is satisfied. In the case of satisfying the two conditions (C1 and C2) given below, the alternative *a*′ is the first best alternative in the $Q$ ranking list.

C1. Acceptable advantage: $Q\left( {a^{\prime\prime}} \right) {-} Q\left( {a^{\prime}} \right) \ge DQ$ , where *a*′′ is the second best alternative in the $Q$ ranking list; $DQ = 1/\left( {m {-} 1} \right)$; where $m$ is the alternatives number.

C2. Acceptable stability: alternative *a*′ must also be the best ranked by $S or R$.

And according to results of applying these two conditions, the judge rules will be as following:-

1. When the second and first ranked alternatives fulfill both C1 and C2, the first ranked alternative is the best alternative.
2. When the second and first ranked alternatives fail only to satisfy C2, the first and second-ranked alternatives are the best alternatives simultaneously.
3. If the condition C1 is not satisfied alternatives $a^{\prime}, a\prime \prime , \ldots , a^{\left( k \right)}$ are determined by the relation $Q\left( {a^{\left( k \right)} } \right) {-} Q\left( {a^{\prime}} \right) \approx DQ$, the positions of these alternatives are “in closeness”^24^.

## Applying the proposed research methodology on the measurements results of the NRR electrical system

Assessment of nuclear facility electrical system from the view of PQ is usually based on studying different PQ phenomena. Among the most important PQ phenomenon, as discussed in “Analysis of NRR power quality problems” section, are (F_dev), (V_Un), (I_Un), (VTHD), (ITHD), (P_lt_), (P_st_), and (PF). Table 2 shows the average of measured PQ divided into six samples. This work aims to select the optimal alternative among six alternatives for different PQ phenomena readings. To do so, the CRITIC, MW, and EW methods are used to calculate the weight of each criterion, then VIKOR and TOPSIS MCDM methods are used for ranking the alternatives and the results of each methodology are presented and discussed. All the mathematical operations of this study were done on Matlab/Simulink software.

**Table 2:** The measured PQ data (Decision matrix).

Alternatives	F_dev	V_Un	I_Un	P_st_	P_lt_	VTHD	ITHD	PF
Sample 1	0.14508	0.0935	3.2588	0.105193333	0.085046667	0.844866667	5.8457	0.9684
Sample 2	0.25958	0.065	4.2029	0.105323333	0.109903333	0.965	8.030833333	0.9747
Sample 3	0.15946	0.0323	3.1262	0.126253333	0.158126667	0.967466667	7.130566667	0.964
Sample 4	0.18056	0.0708	2.8682	0.106096667	0.142603333	1.2088	6.127033333	0.9661
Sample 5	0.31088	0.0739	3.6941	0.097556667	0.10393	0.908266667	6.840866667	0.9648
Sample 6	0.26864	0.0323	2.0803	0.13881	0.20178	0.9539	5.280833333	0.9523

### Weighting for criteria

#### CRITIC weighting method

The original data in Table 2 which presents the decision matrix is normalized and the standard deviation is calculated, as shown in Table 3, based on Eqs. (4–5). Then the correlation coefficient is obtained, as shown in Table 4, based on Eq. (6). After this the weight for each criterion is calculated using Eqs. (7–8) as shown in Table 5.

**Table 3:** Normalized Decision matrix based on CRITIC method.

Alternatives	F_dev	V_Un	I_Un	Pst	Plt	VTHD	ITHD	PF
Sample 1	1	0	0.4448	0.8149	1	1	0.7946	0.7188
Sample 2	0.3094	0.4657	0	0.8117	0.7871	0.6699	0	1
Sample 3	0.9133	1	0.5073	0.3044	0.374	0.6631	0.3274	0.5223
Sample 4	0.786	0.3709	0.6288	0.793	0.5069	0	0.6923	0.6161
Sample 5	0	0.3203	0.2397	1	0.8382	0.8258	0.4327	0.558
Sample 6	0.2548	1	1	0	0	0.7004	1	0
${\upsigma }_{\mathrm{j}}$	0.4093	0.399	0.3412	0.3829	0.3661	0.3402	0.36	0.3276

**Table 4:** Values of criteria correlation coefficient.

	F_dev	V_Un	I_Un	Pst	Plt	VTHD	ITHD	PF
F_dev	1	− 0.1389	0.1761	− 0.0743	0.1021	− 0.1548	0.1716	0.1967
V_Un	− 0.1389	1	0.4896	− 0.8673	− 0.887	− 0.1334	0.02	− 0.6146
I_Un	0.1761	0.4896	1	− 0.7584	− 0.7911	− 0.232	0.8665	− 0.8941
Pst	− 0.0743	− 0.8673	− 0.7584	1	0.9013	− 0.0138	− 0.4156	0.7707
Plt	0.1021	− 0.887	− 0.7911	0.9013	1	0.3408	− 0.4048	0.8098
VTHD	− 0.1548	− 0.1334	− 0.232	− 0.0138	0.3408	1	− 0.0311	− 0.0015
ITHD	0.1716	0.02	0.8665	− 0.4156	− 0.4048	− 0.0311	1	− 0.7431
PF	0.1967	− 0.6146	− 0.8941	0.7707	0.8098	− 0.0015	− 0.7431	1

**Table 5:** Criteria weights.

	F_dev	V_Un	I_Un	Pst	Plt	VTHD	ITHD	PF
${\mathrm{C}}_{\mathrm{j}}$	2.7508	3.6436	2.7786	2.8555	2.5365	2.4583	2.713	2.4488
${\mathrm{W}}_{\mathrm{j}}$	0.124	0.1642	0.1252	0.1287	0.1143	0.1108	0.1223	0.1104

#### Evaluation of obtained weights

The obtained weights based on CRITIC method are evaluated and compared with two other MCDM methods which are; Entropy Weight (EW) method and the Mean Weight (MW) method. The EW method is an objective method that is used to identify the objective weight of each criterion independent of subjective factors. The MW method identifies the criteria objective weight by ${W}_{j}=\frac{1}{n}$ where n is the number of criteria. This is based on the assumption that all criteria have the same importance. It is used in MCDM when information is not sufficient to reach a decision or there is no information from decision maker^32^.

**Figure 11:**
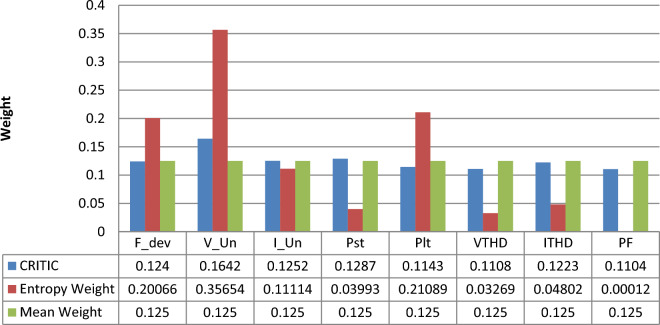
Weight comparison chart.

As shown in Fig. 11, based on MW method all the PQ phenomena take the same weight. While in case of the EW method, the distribution of weight over criteria can reflect the differences between the indicators. It is shown that the voltage unbalance weight is the highest and then long-term flicker followed by frequency deviation. Furthermore, voltage harmonic distortion and power factor have very low importance.

On the other hand, based on the CRITIC method the weight of each PQ index is distributed uniformly giving a higher weight to the voltage unbalance and then short-term flicker followed by current unbalance. Table 6 presents an ordering (ranking) for PQ phenomena based on the obtained weights from both CRITIC and EW objective weighting methods.

**Table 6:** Comparison of PQ phenomena ranking between CRITIC and EW methods.

	F_dev	V_Un	I_Un	P_st_	P_lt_	VTHD	ITHD	PF
CRITIC	5	8	6	7	3	2	4	1
EW	6	8	5	3	7	2	4	1

For three-phase power systems, the occurrence of voltage imbalances can be due to an uneven single phase loads distribution or due to a large single-phase loading^33^. The electrical system of the research reactor under study may have those conditions since it has a lot of single phase loads.

Among the basic safety conditions related to a research reactor are cooling of the reactor core after shutting down the reactor^34^. Accidents or incidents may occur in case of losing reactor coolant pumps which their main function is to remove and transfer the amount of heat generated in the reactor core by providing forced primary coolant flow to the reactor core^35^.This NRR is operating with two core cooling pumps (2000m^3^/h) at its full power (22 MW thermal)^36^ and there are a lot of single phase loads on this facility.

Operating three phase motors (cooling pumps) under voltage unbalance causes problems such as overheating, vibrations, noise, insulation damage, and an increase in line power losses and voltage drop due to the increase in line currents^37^. So that voltage unbalance PQ phenomena presents a great importance to ensure safety for research reactor. Regarding the short-term flicker it has an effect on the reactor shut-down rod which may reduce the availability of the NRR but this still on the acceptable limits where it doesn’t effect on this nuclear facility safety.

This means that all PQ phenomena present a relatively equivalent importance with a higher ranking for the voltage unbalance and short-term flicker from the view of CRITIC method and this is compatible with the safety view of this studied nuclear installation and near to the expert’s opinion.

### Ranking for alternatives

#### VIKOR ranking method

To rank the alternatives, firstly the weighted normalized matrix ${wx}_{new}$ is obtained, as shown in Table 7, based on CRITIC weights and using Eq. (10). Then ${R}_{i}$ and ${\mathrm{S}}_{\mathrm{i}}$ values using Eqs. (11–12) and *Qi* using Eq. (13) are calculated. After this, a three ranking lists of alternatives using ${\mathrm{S}}_{\mathrm{i}}$, ${\mathrm{R}}_{\mathrm{i}}$ and ${\mathrm{Q}}_{\mathrm{i}}$ (for *v* = 0.50) values are determined and sorted as shown in Table 8.

**Table 7:** The weighted normalized matrix.

Alternatives	F_dev	V_Un	I_Un	Pst	Plt	VTHD	ITHD	PF
Sample 1	0	0.1642	0.0695	0.0238	0	0	0.0251	0.031
Sample 2	0.0856	0.0878	0.1252	0.0242	0.0243	0.0366	0.1223	0
Sample 3	0.0108	0	0.0617	0.0895	0.0716	0.0373	0.0823	0.0527
Sample 4	0.0265	0.1033	0.0465	0.0266	0.0564	0.1108	0.0376	0.0424
Sample 5	0.124	0.1116	0.0952	0	0.0185	0.0193	0.0694	0.0488
Sample 6	0.0924	0	0	0.1287	0.1143	0.0332	0	0.1104

**Table 8:** The combined CRITIC-VIKOR method (*v* = 0.5) results.

	${\mathrm{S}}_{\mathrm{i}}$	Rank based on ${\mathrm{S}}_{\mathrm{i}}$	${\mathrm{R}}_{\mathrm{i}}$	Rank based on ${\mathrm{R}}_{\mathrm{i}}$	${\mathrm{Q}}_{\mathrm{i}}$	Rank based on ${\mathrm{Q}}_{\mathrm{i}}$
Sample 1	0.3138	1	0.1642	6	0.5	3
Sample 2	0.5061	6	0.1252	5	0.738955823	6
Sample 3	0.4059	2	0.0895	1	0.239469579	1
Sample 4	0.4502	3	0.1108	2	0.497224467	2
Sample 5	0.4868	5	0.124	3	0.680741687	4
Sample 6	0.479	4	0.1287	4	0.691920046	5

By applying the two conditions of VIKOR method as explained on step 5 of “VIKOR method” section it is found that:

For condition 1 (Acceptable advantage)

$$\begin{aligned} & DQ{ } = { }1/\left( {m{ }{-}{ }1} \right) \\ & DQ = 0.2 \\ & Q\left( {a^{\prime}} \right) = 0.239469579,{ }Q\left( {a^{\prime\prime}} \right) = 0.497224467 \\ & Q\left( {a^{\prime\prime}} \right){ }{-}{ }Q\left( {a^{\prime}} \right) = 0.25775 > { }DQ \\ \end{aligned}$$

that condition 1 is satisfied.

For condition 2 (Acceptable stability).

The second condition is satisfied as the sample 3 is the best alternative and also it is ranked by $R$ as the best one among others alternatives.

As conditions 1 and 2 are satisfied so that this ranking is compromise solution for this MCDM problem.

#### TOPSIS ranking method

In this method (Supplementary File. S1), after normalization of the decision matrix as seen in Table 9, the weighted normalized matrix is built based on the obtained weights from the CRITIC method, as shown in Table 10. The next step is to determine both the Euclidean distance from the ideal best which is called positive ideal solution (**Si+**) and the Euclidean distance from the ideal worst which is called the negative ideal solution (**Si−**), the results is shown in Table 11.

**Table 9:** The normalized matrix.

Alternatives	F_dev	V_Un	I_Un	P_st_	P_lt_	VTHD	ITHD	PF
Sample 1	0.258383	0.5852	0.4065	0.3763	0.2495	0.3515	0.3613	0.4097
Sample 2	0.462304	0.40683	0.5243	0.3768	0.3225	0.4015	0.4964	0.4123
Sample 3	0.2839934	0.20216	0.39	0.4517	0.4639	0.4025	0.4407	0.4078
Sample 4	0.3215718	0.44313	0.3578	0.3795	0.4184	0.5029	0.3787	0.4087
Sample 5	0.5536678	0.46253	0.4608	0.349	0.3049	0.3779	0.4228	0.4081
Sample 6	0.4784396	0.20216	0.2595	0.4966	0.592	0.3969	0.3264	0.4028

**Table 10:** The weighted normalized matrix.

Alternatives	F_dev	V_Un	I_Un	P_st_	P_lt_	VTHD	ITHD	PF
Sample 1	0.032038	0.09611	0.0509	0.0484	0.0285	0.039	0.0442	0.0452
Sample 2	0.057323	0.06682	0.0657	0.0485	0.0369	0.0445	0.0607	0.0455
Sample 3	0.0352135	0.0332	0.0488	0.0581	0.053	0.0446	0.0539	0.045
Sample 4	0.0398731	0.07278	0.0448	0.0489	0.0478	0.0557	0.0463	0.0451
Sample 5	0.0686516	0.07596	0.0577	0.0449	0.0349	0.0419	0.0517	0.0451
Sample 6	0.0593238	0.0332	0.0325	0.0639	0.0677	0.044	0.0399	0.0445

**Table 11:** Calculating (Si +) and (Si-) for each alternative.

Alternatives	Sample 1	Sample 2	Sample 3	Sample 4	Sample 5	Sample 6
Si +	0.0658	0.0584	0.0358	0.0499	0.0632	0.0516
Si-	0.0623	0.048	0.076	0.0513	0.0467	0.0911

Finally, the performance index Pi of each alternative (sample) can be obtained, and depending on Pi values a ranking for alternatives is obtained as shown in Table 12^26^.

**Table 12:** Ranking alternatives by TOPSIS method.

Alternatives	Sample 1	Sample 2	Sample 3	Sample 4	Sample 5	Sample 6
Pi	0.5134	0.5491	0.3201	0.4929	0.575	0.4058
Rank	4	5	1	3	6	2

#### Analysis for ranking results

In the present study a two widely used MCDM methods (VIKOR and TOPSIS) are utilized to assess six alternatives with eight criteria for different PQ phenomenon readings.

**Figure 12:**
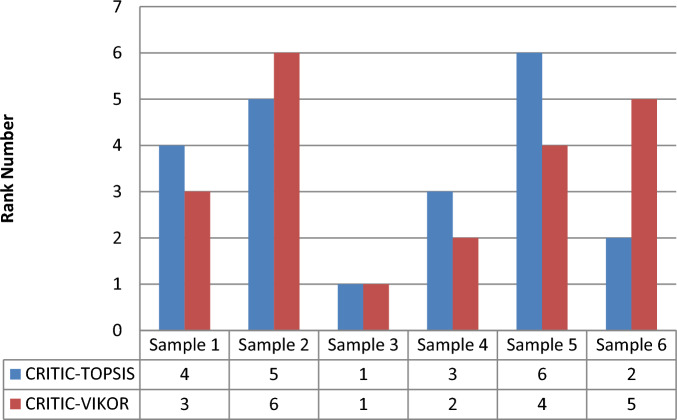
Rank comparison chart.

As shown in Table 13, the results that are obtained by the two methods showed that sample 3 is the best alternative and other alternatives in different positions in the two ranking methods but there is a relative similarity in the ranking with the two methods. Figure 12 presents an ordering (ranking) for the studied samples based on the obtained ranking values from both TOPSIS and VIKOR.

**Table 13:** Ranking comparison between TOPSIS and VIKOR.

Alternatives	TOPSIS		VIKOR	
	Value	Ranks	Value	Ranks
Sample 1	0.51344	4	0.5	3
Sample 2	0.54913	5	0.738955823	6
Sample 3	0.32009	1	0.239469579	1
Sample 4	0.49288	3	0.497224467	2
Sample 5	0.57504	6	0.680741687	4
Sample 6	0.40579	2	0.691920046	5

## Conclusion

This study aims to assess performance of PQ for the electrical power system at a Nuclear Research Reactor (NRR) during a certain period using multiple measures for the most decisive PQ phenomena. It focuses on a number of the most important PQ phenomena namely frequency fluctuation (deviation), unbalances of current and voltage, current and voltage harmonic distortion, flicker and power factor.

Since the decision problem of this study depends on multi-criteria and a number of alternatives, so that a MCDM methodologies is used. Among the MCDM methods is the CRITIC method which is used for weighting and has the advantages of strong operability and less calculation compared with the other objective and subjective weighting methods. Also, VIKOR ranking method is very simple and robust because it contains smaller number of steps for calculating the ranking of each alternative.

Targeting to the effect of the correlation between the performances of PQ phenomena on the PQ evaluation results, this study established an evaluation model by combining the CRITIC-VIKOR methods. This combination is appropriate for a variety of assessment scenarios, mainly for targets with multiple indices.

Using the measurements of the different mentioned PQ phenomena for NRR electrical system, objective weights for these PQ phenomena (criteria) are determined to be much more appropriate than subjective weights. By analyzing the obtained weights and comparing them with other methodology, it is found that the weights based on CRITIC method compatible with the safety view of this studied nuclear installation and near to the expert’s opinion. And this reflects the realists of this methodology. Moreover, the VIKOR ranking results are compared with another evaluation result and showed the superiority and validity of VIKOR method for comprehensive assessment of PQ on the used nuclear installation.

These used methodologies can not only reflect the correlation between indicators, weaknesses and strengths of PQ, but also can be applied to present an evaluation of PQ in real time according to the actual operation of the loads in the nuclear installation. If it is applied with on-line measurements, it can be more follow the actual operation of the nuclear facility, which presents a good application in the PQ management for this type of critical installations.

A more research can be done by inserting the renewable energy on this electrical system and showing the PQ performance changes. Also, more advanced techniques in MCDM field can be proposed and applied on the nuclear installation power system and other electrical networks to assess and evaluate the total PQ performance.

## Supplementary Information

Supplementary Information.

## Data Availability

The datasets used and/or analysed during the current study available from the corresponding author on reasonable request.
