# Supplementary material for: Comprehensive power quality performance assessment for electrical system of a nuclear research reactor
Source: Sci Rep. 2023 Jun 19;13:9915. doi: 10.1038/s41598-023-36692-2 (PMC10279760; doi:10.1038/s41598-023-36692-2)
Supplement: Supplementary file 1 — Supplementary Information. [file 41598_2023_36692_MOESM1_ESM.docx]

**Supplementary material**

Comprehensive Power Quality Performance Assessment for Electrical System of a Nuclear Research Reactor

Asmaa M. Elsotohy^1🖂^, Ahmed Mohammed Attiya Soliman^2^, Ahmed S. Adail^3^, Ayman A. Eisa^1^& El-said A Othman^2^

^1^Department of Nuclear Safety and Radiological Emergencies, NCRRT , Egyptian Atomic Energy Authority, Egypt. ^2^Department of Electrical Engineering, Faculty of Engineering, Al-Azhar University, Cairo, Egypt. ^3^Department of Fuel Technology, Hot laboratory Centre, Egyptian Atomic Energy Authority, Egypt. ^🖂^[Asmaa.sotohy@eaea.org.eg](mailto:Asmaa.sotohy@eaea.org.eg)

**TOPSIS Ranking Method**

The technique for order of preference by similarity to ideal solution (TOPSIS) method is one of the best multi-criteria decision-making methods (MCDM) for ranking alternatives. It is based on the concept that the alternatives should have the longest distance from the negative ideal solution-(NIS) and the shortest distance from the positive ideal solution (PIS). The NIS is the solution point with highest cost and lowest benefit, while PIS is the solution point with lowest cost and highest benefit.

To apply this method to MCDM problems, we can follow the steps in the flowchart in Figure [S1](#_bookmark9).

Step1: Vector normalization for the decision matrix

Step2: Establish the weighted normalized matrix

Step3: Determine the positive and negative ideal solution

Step 4: Calculating closeness of each alternative to the ideal solution and ranking the alternatives

**Figure S1.** Flowchart of the implementation steps of the TOPSIS method.

**The ranking algorithm TOPSIS has the following steps:-**

**Step 1:** Normalize the decision matrix after calculating the normalized value $n_{ij}$ for each criteria relating to all alternatives using Equ. S1.

| (S1) | $n_{ij}=\frac{a_{ij}}{\sqrt{\sum_{i=1}^{m} {a_{ij}}^{2}}}$ |
| --- | --- |
|  | *i=*$i = 1, 2, \ldots, m$ _For m alternatives_ |
|  | $j = 1, 2, \ldots, n$ _For n criteria_ |

Where, $a_{ij}$ is the value of $j_{th}$ criterion for the alternative $a_{i}$

**Step 2:** Computing the weighted normalized matrix

Using the weight of each criterion $w_{j}$ which is calculated based on objective or subjective weighting method, the weighted normalized matrix is built.

**Step3:** Calculate the Euclidean distance from the ideal best ($s_{i}^{+}$) and Euclidean distance from the ideal worst ($s_{i}^{-}$) using Equs. (S2, S3)

| $s_{i}^{+}={[\sum_{j=1}^{n} \left( V_{ij}-V_{j}^{+} \right)^{2}]}^{0.5}$ | (S2) |
| --- | --- |
| $s_{i}^{-}={[\sum_{j=1}^{n} \left( V_{ij}-V_{j}^{-} \right)^{2}]}^{0.5}$ | (S3) |

Where $s_{i}^{+}$ indicates the ideal positive solution that represents the least possible risk, and $s_{i}^{-}$ indicates the ideal negative solution that represents the highest possible risk.

$V_{ij}$ is the value of $j_{th}$ criterion for the alternative $a_{i}$, $V_{j}^{+}$ the best value for each criteria and $V_{j}^{-}$ the worst value for each criteria in the normalized decision matrix

**Step 4:** Calculating the performance score $P_{i}$ using Equ. S4

| $P_{i}=\frac{s_{i}^{-}}{s_{i}^{+}+s_{i}^{-}}$ | (S4) |
| --- | --- |

The performance score $P_{i}$ is an indicator for Relative Closeness of each alternative to the Ideal Solution

**Matlab Code for Weighting and Ranking MCDM Methodologies**

Entropy Weight Method Matlab Code


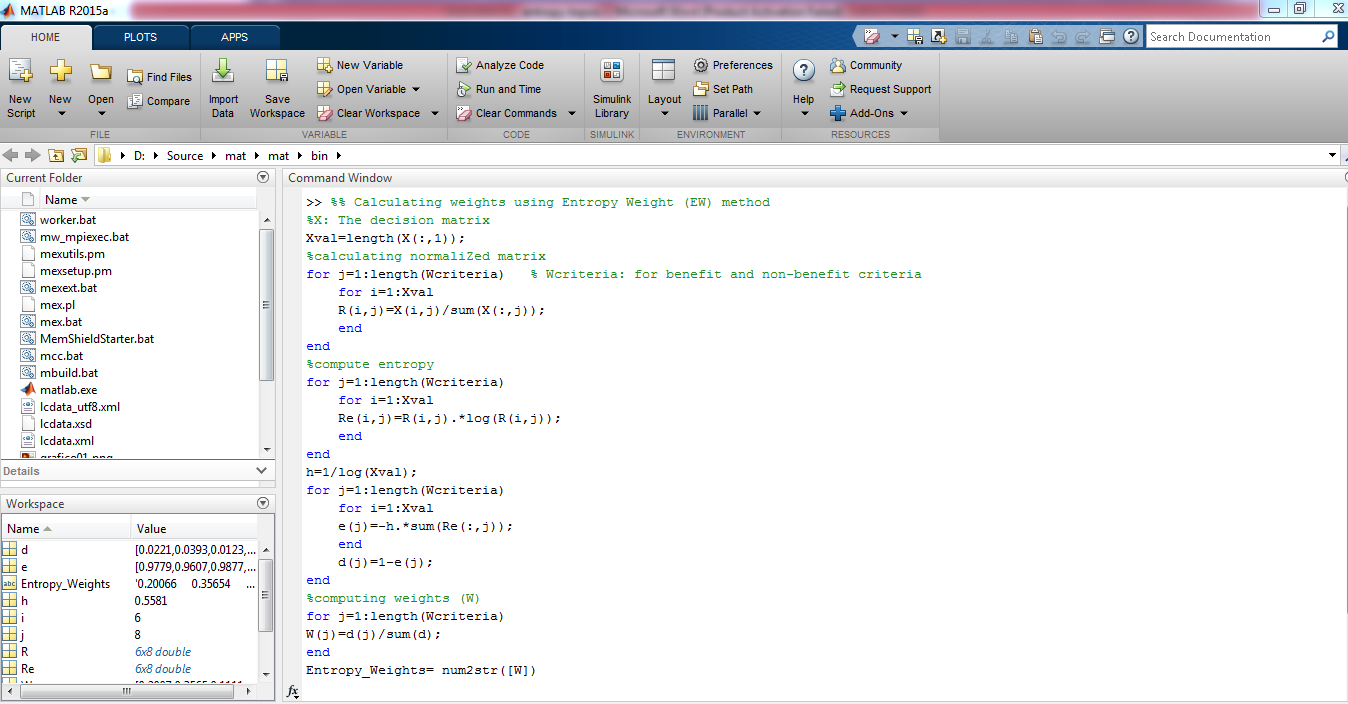


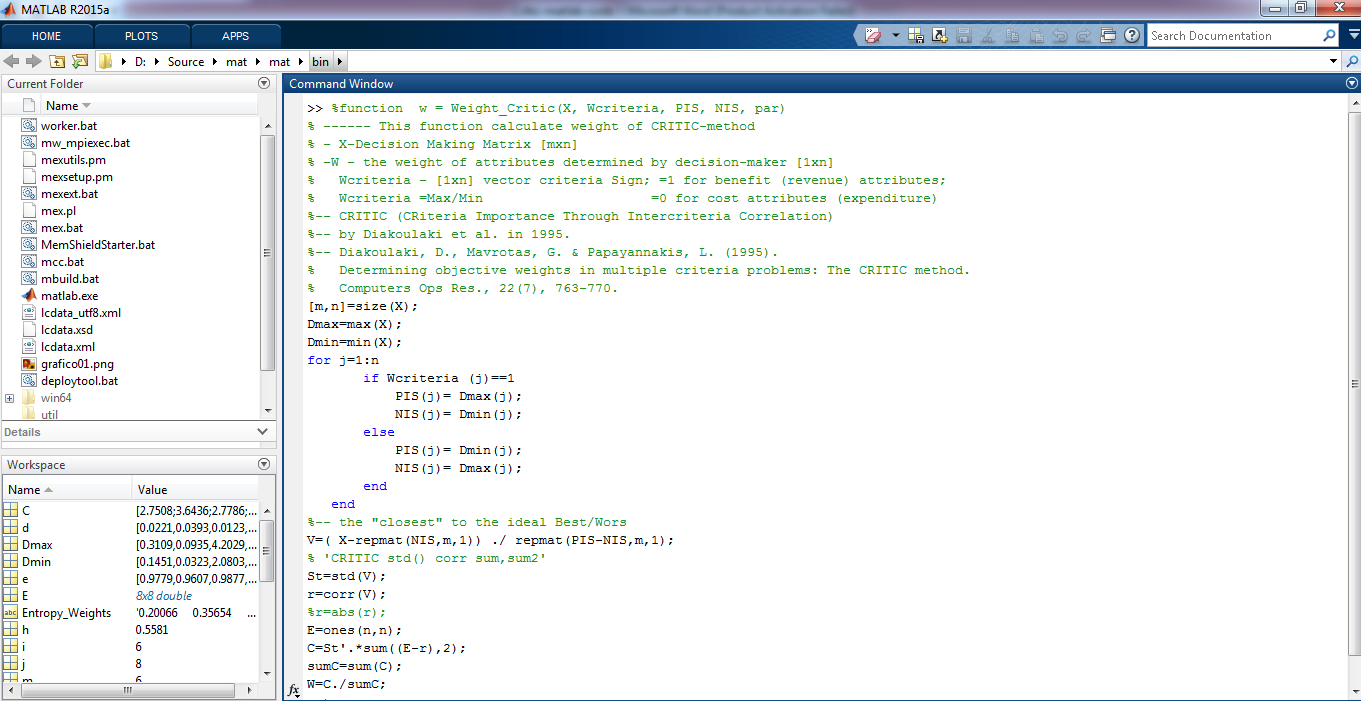


TOPSIS Ranking Method Matlab Code


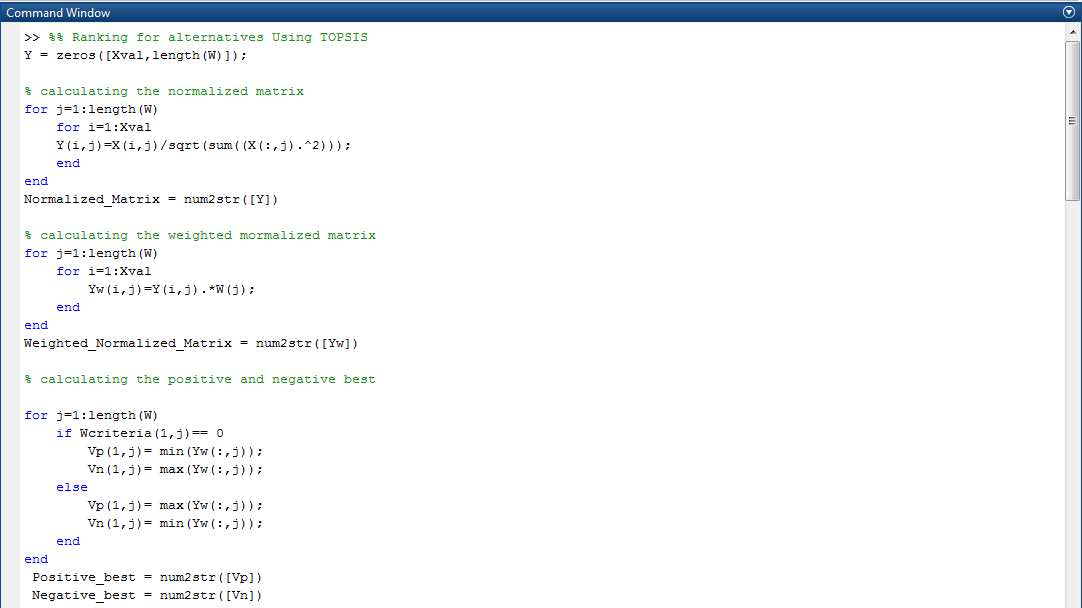


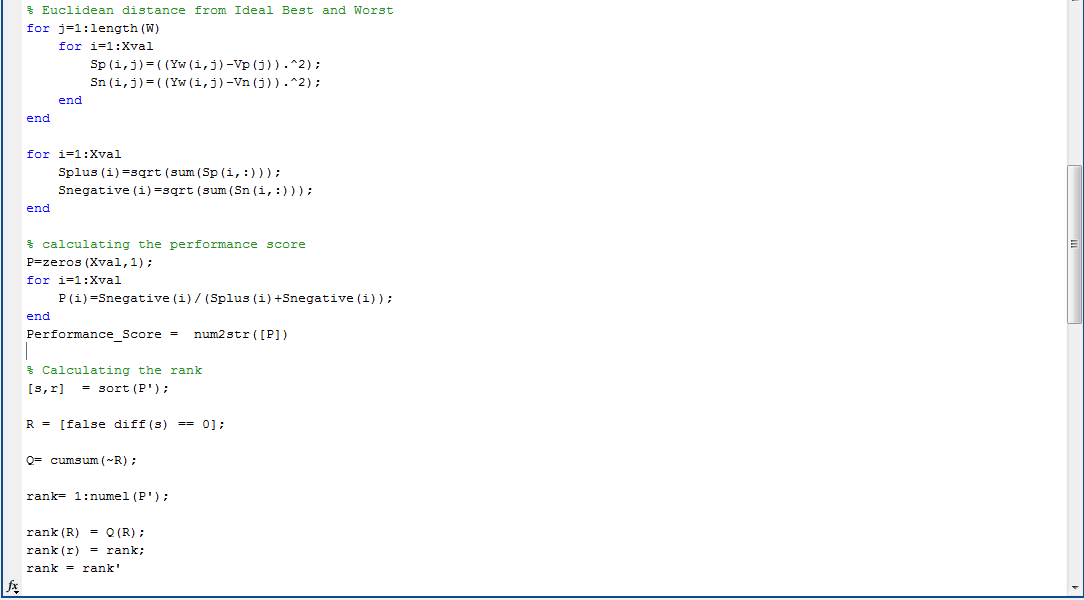


VIKOR ranking method Matlab Code


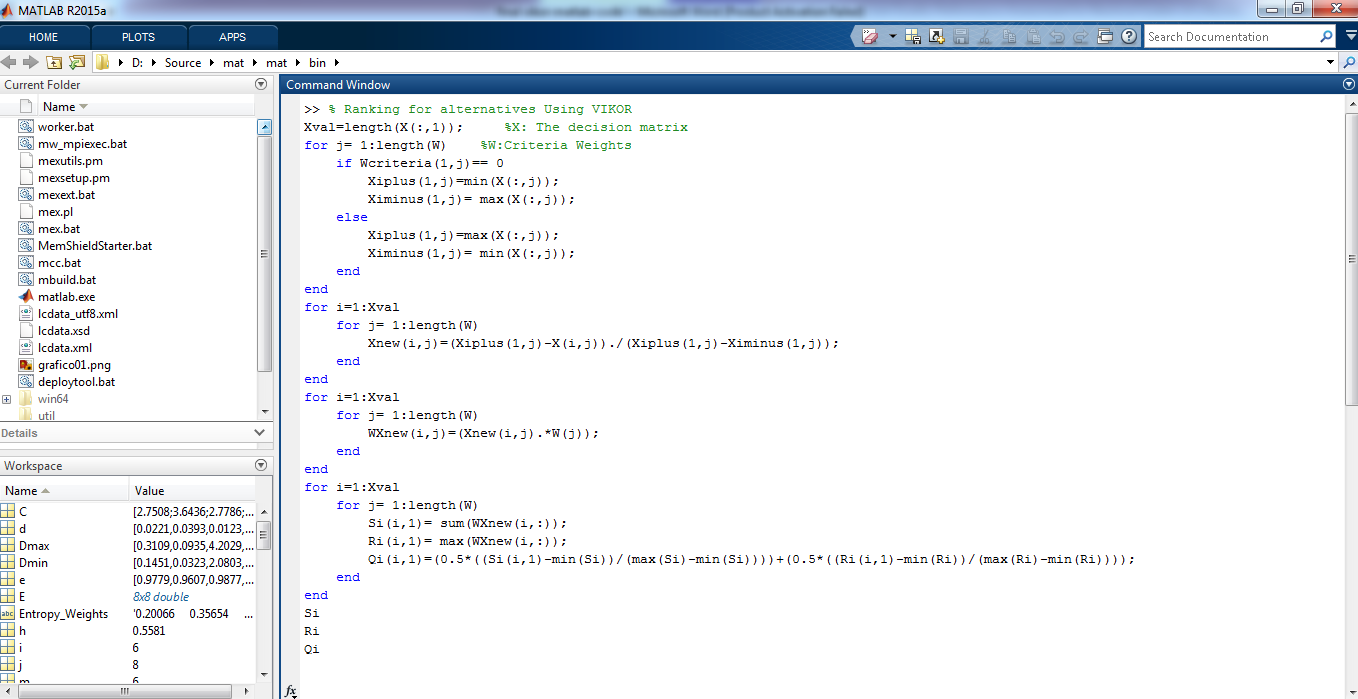


*The information in this supplementary file presents a more details and explaining for results on the attached main original paper "Comprehensive Power Quality Performance Assessment for Electrical System of a Nuclear Research Reactor"
